# Oral *Streptococcus mutans* load among Indian children with cerebral palsy

**DOI:** 10.6026/97320630019215

**Published:** 2023-02-28

**Authors:** Aniruddh Gandhi, Subhash Sonkesriya, Shovan Roy, Raman Mishra, Jatin Arora, Vineet Soni

**Affiliations:** 1 Department of Dentistry, Late Shri Atal Bihari Vajpayee Memorial Government Medical College, Rajnandgaon, Chhattisgarh, India; 2 Department of prosthodontics Government College of Dentistry Indore, M.P., India; 3 Department of Orthodontics and Dentofacial Orthopedics, Hazaribagh College of Dental Sciences and Hospital, Hazaribagh, Jharkhand, India; 4 Department of Conservative Dentistry and Endodontics Sharda School of Dental Sciences, Sharda University, Knowledge Park III, Greater Noida, Uttar Pradesh, India; 5 Clinical Practionner in Mumbai, Maharashtra, India; 6 Department of Periodontology and Oral Implantology, Daswani Dental College and Research Centre, Ranpur, Kota, Rajasthan, India

**Keywords:** Oral *Streptococcus mutans*, *cerebral palsy*

## Abstract

The motor impairments of cerebral palsy (CP) are typically accompanied by subsequent musculoskeletal issues, seizures, and abnormalities of sensation, intelligence, communication, and behaviour. These kids have a lower capacity for regulating oral health because of their poor voluntary movements. Poor oral hygiene brought on by insufficient brushing and flossing, increased use of sugary foods, and orally administered drugs puts people at risk for periodontal disorders and dental caries. Poor dental health and rising therapy demands establish a sadistic cycle that affects patient overall health and wellbeing. The purpose of this investigation was comparing kids with CP against healthy kids of comparable age group and demographic situation in order to evaluate status of oral heath, current caries behavior using measurement of *Streptococcus mutans* concentrations in saliva, and treatment required. 204 study participants were divided into two categories: Category A and category B. Both categories consisted of 102 study participants. Category A consisted of study participants having CP while category B consisted of healthy normal controls with same age of same demographic features. Malocclusion, trauma, DMFS/defs, gingival index, and Oral hygiene score (OHI), and were recorded for oral examinations of al study participants However, no radiological assistance was utilized since minimal patient compliance existed in CP patients. When compared with the control category, the CP category had a higher detection of the DMFS index in the permanent teeth. The estimated defs for the CP category did not differ noticeably from the control category. In the CP category, status of hygiene of oral cavity was discovered to be substantially subpar. In comparison to the control category, the gingival condition of the CP category was noticeably worse. Treatment requirements were seen to require greater preventative care in the control category while, stainless steel crowns, pulpectomy and extractions were needed in the CP category. *S. mutans* was found in high concentrations in the salivary specimens of the CP category compared to the control category, indicating active dental caries and greater probability of further development.

## Background:

Cerebral palsy (CP) is a collection of disorders in which cognitive and physical incapacity result in a dearth of neuromuscular synchronization to carry out everyday tasks and an inability to comprehend necessary dental care in those affected. The motor impairments of CP are typically accompanied by subsequent musculoskeletal issues, seizures, and abnormalities of sensation, intelligence, communication, and behaviour. [1] These kids have a lower capacity for regulating oral health because of their poor voluntary movements. [2] Poor oral hygiene brought on by insufficient brushing and flossing, increased use of sugary foods, and orally administered drugs puts people at risk for periodontal disorders and dental caries. [3] Poor dental health and rising therapy demands establish a sadistic cycle that affects patient overall health and wellbeing. Despite enormous improvements in treatment and prevention tooth decay is still a very common disease [4, 5, 6, 7, 8, 9, 10, 11, 12, 13, 14, 15, 16,17]. Numerous factors interact to affect the progression and growth of caries, according to research. The biological assumption that variations in salivary characteristics may influence the emergence of dental decay has been examined in several in vitro investigations [5, 18]. However, there is disagreement regarding the prevalence of dental decay and the salivary factors related to it in kids with CP [19]. When distributing funds for the preventative treatment of caries, it is crucial to identify people who are at a significant risk of developing dental problems as well as children who have unique health requirements. Underappreciated hygiene of oral cavity may contribute to dietary deficits such protein energy deprivation, endocrine disorders, and impaired growth as shown in the appearance of slowed steady growth, losing weight, aberrant anthropometric and diminished bone strength. [4] It emphasizes the significance of ongoing therapy demands monitoring and quick response to preserve both dental health and general wellbeing in CP affected patients. Therefore, the purpose of this investigation was comparing kids with CP against healthy kids of comparable age group and demographic situation in order to evaluate status of oral heath, current caries behavior using measurement of *Streptococcus mutans* concentrations in saliva, and treatment required.

## Materials and Methods:

In order to prevent errors caused by investigator misreading, the research begins with standardizing the test to be completed by a solitary investigator under inspection and randomized evaluation of data collected. The criteria for inclusion of a child with a confirmed professional specific diagnosis of CP guided the identification of subjects. [5] Patients who received any surgical procedure intended to restrict salivary flow were excluded from the research. [6]

## Sample size:

n = (z)^2^ p (1 - p) / d^2^

n = sample size

z = level of confidence according to the standard normal distribution (for a level of confidence of 95%, z = 1.25

p = estimated proportion of the population that presents the characteristic (when unknown we use p = 0.5)

d = tolerated margin of error (for example we want to know the real proportion within 5%)

Using above formula, a minimum sample size of 208 was calculated These 208 study participants were divided into two categories: Category A and category B. Both categories consisted of 102 study participants. Category A consisted of study participants having CP while category B consisted of healthy normal controls with same age of same demographic features. Malocclusion, trauma, DMFS/defs, gingival index, and Oral hygiene score (OHI), and were recorded for oral examinations of al study participants However, no radiological assistance was utilized since minimal patient compliance existed in CP patients.

## Microbiology assessment:

The following were subjected to microbiological standardization: (1) development of culture (MSB agar); placement of specimen in culture medium and assessment of *S. mutans* colony architecture. After indices evaluation, three ml of saliva specimen was taken from the kids who had been inspected after they were instructed to cleanse their mouths to remove food particles and agglomerated cells. The youngster was asked to sit down having their head a little bit lowered while spit was being collected in order to retrieve unaroused saliva. The oral cavity floor was left to gather saliva productions, and 50 kids who really can follow directions were instructed to spew into a clean graduated container once three ml of saliva was obtained. The remaining 54 kids, who struggled to follow instructions, had samples taken from their mouths and put in sample collecting vials using a sterile micropipette. Following that, the material was taken to a science laboratory for colony cultivation and characterization. A single inoculum of salivary sample was seeded onto MSB agar, immediately. The inoculated culture plates were incubated for duration of 48 hours in an aerotolerant atmosphere using a CO2 incubator (five percentage CO2 at temperature of 37°C). [14] *S. mutans* colonies were recognized by their distinctive colony traits. *S. mutans* colony numbers were quantified and isolated using a quantitative culture approach. (CFU)/ml, were used to represent the microbiological quantities.

## Statistical analysis:

The SPSS programme version 20 was used to examine the data. According to the different types of variables being researched, the Student's t test, Mann-Whitney test and Chi square test had been incorporated, with the degree of significance being P value less than 0.05.

## Results:

In this study each category consisted of 104 study participants. Category A consisted of 104 children affected by CP while category B consisted of normal healthy subjects with same demographic details. Mean age in category A study participants was 7.89±1.731 years while Mean age in category B study participants was 6.58±1.156 years. The male study participants in category A were 81(77.89%) while the female study participants were 23 (23.11%). The male study participants in category B were 60 (57.69%) while the female study participants were 54 (42.31%). The frequency of males was greater in category A i.e CP affected children (Table 1).

The mean DMFS values for permanent teeth in study participants in category A was 1.08±3.16 while the mean DMFS values in permanent teeth for participants in category B was 0.14±0.76. The values in permanent dentition were greater in study participants affected with CP as compared to control group children showing that decaying of teeth is more common in CP influenced children. The findings were significant statistically with p value = 0.0001. The mean defs values for deciduous teeth in participants in category A was 8.06±14.22 while the mean defs values in deciduous teeth for participants in category B was 4.50±7.11. The values in deciduous dentition were greater in study participants affected with CP as compared to control group children showing that decaying of teeth is more common in CP influenced children. However the findings were non- significant statistically with p value = 0.099. The mean GI values was 1.14±0.86 in study participants of category A while mean GI values was 0.35±0.58 in study participants of category B. The values were greater in children affected with CP as compared to control group children showing poor gingival health in CP affected kids. The p value was 0.0001 showing that the differences were vital statistically. The mean OHI values was 8.02±1.45 in study participants of category A while mean OHI values was 7.07±0.54 in study participants of category B. The values were greater in children affected with CP as compared to control group children showing poor oral hygiene in CP affected kids. The p value was 0.0001 showing that the differences were vital statistically (Table 2).

When there was evaluation of injury then it was observed that no incidence of injury or fracture was observed in 95.3% study participants in category A while such condition was observed in 97.4% study participants in category B. It was observed that Ellis class IX fracture was observed in 2.7% study participants in category A while such condition was observed in 2.6% study participants in category B. It was observed that Ellis class I fracture was observed in 2.0% study participants in category A while no such condition was observed in study participants in category B. The findings were comparable statistically with p=0.34. It showed that incidence of trauma was comparable in both category study participants. (Table 3)

In this study, flush terminal plane was present in 2 (1.9%) of study participants in category A, while it was 2 (1.9%) of study participants in category B. Mesial step molar relation and distal step molar relation was not observed in in study participants of category A while it was 2 (1.9%) in category B. Class I molar relation was observed in 64 (61.5%) of study participants in category A, while it was 62 (59.6%) of study participants in category B. Class II molar relation was observed in 28 (26.9%) of study participants in category A, while it was 24 (23.1%)of study participants in category B. Class III molar relation was observed in 10 (9.6%) of study participants in category A, while it was 12 (11.5%) of study participants in category B. The differences regarding incidence of malocclusion was not significant statistically. (p=0.71). (Table 4)

It was observed that preventive caries arresting care was more frequently observed in category B (76) when contrasted against category A (28).The difference vital statistically because p= 0.0001. Similarly fissure sealant was more frequently observed in category B (60) when contrasted against category A (28).The difference vital statistically because p= 0.0001. One surface filling was comparability observed in category B (42) when contrasted against category A (38).The difference non-vital statistically because p= 0.42. Two surface filling was comparability observed in category B (42) when contrasted against category A (60).The difference non-vital statistically because p= 0.11. Crown due any cause was more frequently observed in category A (64) when contrasted against category B (40).The difference vital statistically because p= 0.01. Pulp therapy with restoration was more frequently observed in category A (64) when contrasted against category B (34).The difference vital statistically because p= 0.01. Extraction more frequently observed in category A (56) when contrasted against category B (32).The difference vital statistically because p= 0.029. Oral prophylaxis more frequently observed in category A (33) when contrasted against category B (30).The difference vital statistically because p= 0.0001. Orthodontic intervention was comparability observed in category B (42) when contrasted against category A (38).The difference non-vital statistically because p= 0.42. (Table 5)

104 specimens in both category A and B were cultured for analysis of colonies of *S. mutans*. 17 specimens in category A and 13 specimens in category B were found to have 10^6^ -10^5^ CFU/ml. 17 specimens in category A and 2 specimens in category B were found to have 10^5^ -10^4^ CFU/ml. 12 specimens in category A and 24 specimens in category B were found to have 10^4^ -10^3^ CFU/ml. 10 specimens in category A and 17 specimens in category B were found to have no traces of *S. mutans*. The observations were relevant statistically with p=0.001. It was observed that higher quantities of *S. mutans* were observed in CP affected children. (Table 6)

## Discussion:

In people with cerebral palsy (CP), there is a lack of neuromuscular synchronization that makes it difficult for them to do daily duties and to understand why they need to receive basic dental care. Aside from seizures and consequent musculoskeletal problems, the motor impairments of CP are frequently accompanied with anomalies in sensory, IQ, behaviour, and communication. [7, 8] Because of their weak voluntary movements, these children have a decreased potential for controlling oral health. [9,10] People who have poor oral hygiene are more likely to develop periodontal diseases and dental caries, which are brought on by inadequate brushing and flossing, increased consumption of sweet foods, and medicines taken orally. [11, 12, 13] Patients' general health and welfare are impacted by the cruel cycle created by poor tooth health and increasing therapy needs. Poor dental hygiene can lead to food deficiencies including protein energy deficiency, endocrine abnormalities, and stunted growth, as seen by the symptoms of slower steady growth, weight loss, abnormal anthropometrics, and decreased bone strength. [14, 15, 16, 17, 18] The importance of ongoing therapy necessitates monitoring and prompt action is emphasized in order to maintain both oral health and general welfare [18, 19, 20, 21, 22, 23, 24, 25]. To assess the status of oral health, current caries behaviour using assessment of *Streptococcus mutans* concentrations in saliva, and necessary therapy, this inquiry compared kids with CP to healthy kids of comparable age group and demographic circumstances. It was observed that the mean DMFS values for permanent teeth in study participants in category A was 1.08±3.16 while the mean DMFS values in permanent teeth for participants in category B was 0.14±0.76. The values in permanent dentition were greater in study participants affected with CP as compared to control group children showing that decaying of teeth is more common in CP influenced children. The findings were significant statistically with p value = 0.0001. The mean defs values for deciduous teeth in participants in category A was 8.06±14.22 while the mean defs values in decidous teeth for participants in category B was 4.50±7.11. The values in deciduous dentition were greater in study participants affected with CP as compared to control group children showing that decaying of teeth is more common in CP influenced children. However the findings were non- significant statistically with p value = 0.099. Santos et al. also discovered that kids with CP had substantially greater DMFS scores than children without CP (P =0.05). [7] Other research with identical outcomes have suggested that meal consistency, lengthy oral medicine use associated with impaired oral motor coordination skills, and challenges with oral cleanliness are likely causes of increased caries experiences. In this study the mean GI values was 1.14±0.86 in study participants of category A while mean GI values was 0.35±0.58 in study participants of category B. The values were greater in children affected with CP as compared to control group children showing poor gingival health in CP affected kids. The p value was 0.0001 showing that the differences were vital statistically. The mean OHI values was 8.02±1.45 in study participants of category A while mean OHI values was 7.07±0.54 in study participants of category B. The values were greater in children affected with CP as compared to control group children showing poor oral hygienr in CP affected kids. The p value was 0.0001 showing that the differences were vital statistically. Similar observations were noticed by Sinha et al [2] who found poor oral hygiene and poor gingival health in CP affected children. When there was evaluation of injury then it was observed that no incidence of injury or fracture was observed in 95.3% study participants in category A while such condition was observed in 97.4% study participants in category B. It was observed that Ellis class IX fracture was observed in 2.7% study participants in category A while such condition was observed in 2.6% study participants in category B. It was observed that Ellis class I fracture was observed in 2.0% study participants in category A while no such condition was observed in study participants in category B. The findings were comparable statistically with p=0.34. It showed that incidence of trauma was comparable in both category study participants. In previous research by Holan et al [25] the findings obtained were not in accordance with the present research because they observed increased frequency of trauma to maxillary anterior teeth in CP affected children. In this study, flush terminal plane was present in 2 (1.9%) of study participants in category A, while it was 2 (1.9%) of study participants in category B. Mesial step molar relation and distal step molar relation was not observed in in study participants of category A while it was 2 (1.9%) in category B. Class I molar relation was observed in 64 (61.5%) of study participants in category A, while it was 62 (59.6%) of study participants in category B. Class II molar relation was observed in 28 (26.9%) of study participants in category A, while it was 24 (23.1%)of study participants in category B. Class III molar relation was observed in 10 (9.6%) of study participants in category A, while it was 12 (11.5%) of study participants in category B. The differences regarding incidence of malocclusion was not significant statistically. (p=0.71). There were some previous studies like Huang et al [24], Santos et al [7] which showed similar results. Crown due any cause was more frequently needed in category A (64) when contrasted against category B (40).The difference vital statistically because p= 0.01. Pulp therapy with restoration was more frequently needed in category A (64) when contrasted against category B (34).The difference vital statistically because p= 0.01. Extraction more frequently needed in category A (56) when contrasted against category B (32).The difference vital statistically because p= 0.029. Oral prophylaxis more frequently observed in category. It was observed that need for different dental treatments were greater in CP affected kids as contrasted against normal controls.

Similarly Huang et al. reported that most of the treatment needs in CP children escalate with increasing age.[24] In this study 104 specimens in both category A and B were cultured for analysis of colonies of *S. mutans*. 17 specimens in category A and 13 specimens in category B were found to have 106 -105 CFU/ml. 17 specimens in category A and 2 specimens in category B were found to have 10^5^ -10^4^ CFU/ml. 12 specimens in category A and 24 specimens in category B were found to have 10^4^ -10^3^ CFU/ml. 10 specimens in category A and 17 specimens in category B were found to have no traces of *S. mutans*. The observations were relevant statistically with p=0.001. It was observed that higher quantities of *S. mutans* were observed in CP affected children. A study was conducted by Jordan et al [23] and he also observed that increased levels of *S. mutans* were observed in CP affected children. They also found a positive correlation between caries activity and levels of *S. mutans*. The goal of the current study was to compare and contrast between the overall oral well-being of kids with CP and kids of similar age in order to highlight the subtle distinctions between the two groups and show how the dental needs of special needs children differ from those of a typically developing young child. However, a couple of the study's weaknesses included the absence of radiographic examinations of both groups to understand the amount of carious lesions and any treatment interventions for maintaining oral hygiene and controlling caries in both groups.

## Conclusion:

When compared with the control category, the CP category had a higher detection of the DMFS index in the permanent teeth. The estimated defs for the CP category did not differ noticeably from the control category. In the CP category, status of hygiene of oral cavity was discovered to be substantially subpar. In comparison to the control category, the gingival condition of the CP category was noticeably worse. Treatment requirements were seen to require greater preventative care in the control category while, stainless steel crowns, pulpectomy and extractions were needed in the CP category. *S. mutans* was found in greater concentrations in the salivary specimens of the CP category compared to the control category, indicating active dental caries and greater probability of further development.

## Figures and Tables

**Table 1:** Data regarding demographic feature in category A and category B

**Variables**	**Sample size (n=208)**	**Age group (mean age group)**	**Gender**	
			**Male**	**Female**
Category A	104	7.89±1.731 years	81(77.89%)	23 (23.11%)
Category B	104	6.58±1.156 years	60 (57.69%)	54 (42.31%)

**Table 2:** Data regarding OHI values, GI values, defs values and DMFS values.

	**Mean DMFS**	**Mean defs**	**Mean GI**	**Mean OHI**
Category A	1.08±3.16	8.06±14.22	1.14±0.86	8.02±1.45
Category B	0.14±0.76	4.50±7.11	0.35±0.58	7.07±0.54
P (Mann-Whitney U test)	0.0001	0.099	0.0001	0.0001
Significance	S	NS	S	S

**Table 3:** Data regarding trauma to teeth

**Assessmentofinjury**	**Noinjuryorfracture**	**EllisclassIXfracture**	**EllisclassIfracture**	**Pvalue**
CategoryA(n=104)	95.30%	2.70%	2	0.34
CategoryB(n=104)	97.40%	2.60%	0	

**Table 4:** Data regarding malocclusion in category A and category B

**Molar relation**	**Flush terminal molar relation**	**Mesial step molar relation**	**Distal step molar relation**	**Class I molar relation**	**Class II molar relation**	**Class III molar relation**	**P value**
Category A	2 (1.9%)	0	0	64 (61.5%)	28 (26.9%)	10 (9.6%)	0.71
							
Category B	2 (1.9%)	2 (1.9%)	2 (1.9%)	62 (59.6%)	24 (23.1%)	12 (11.5%)	

**Table 5:** Data regarding different dental treatments in category A and category B

	**Category B (n=104)**	**Category A (n=104)**	**P' value (Mann-whitney U test)**
Preventive caries arresting care	76	28	0.0001
Fissure sealant	60	28	0.0001
One surface filling	42	38	0.42
Two surface filling	42	60	0.11
Crown due any cause	40	64	0.01
Veneer or laminate	0	0	0
Pulp therapy with restoration	34	64	0.01
Extraction	32	56	0.029
Oral prophylaxis	30	33	0.0001
Orthodontic intervention	6	6	1
Not recorded	0	0	0

**Table 6:** Data regarding CFU/ml of *Streptococcus mutans* species

	**Total number of specimens cultured for species of *Streptococcus mutans***	**Colony count (CFU/ml) of species of *Streptococcus mutans***				
		106 -105	105 -104	104 -103	Not detectable	P value
Category A (n=104)	104	17	17	12	10	0.001
						
Category B (n=104)	104	13	2	24	17	
